# T-817MA, but Not Haloperidol and Risperidone, Restores Parvalbumin-Positive **γ**-Aminobutyric Acid Neurons in the Prefrontal Cortex and Hippocampus of Rats Transiently Exposed to MK-801 at the Neonatal Period

**DOI:** 10.5402/2012/947149

**Published:** 2012-07-08

**Authors:** Takashi Uehara, Tomiki Sumiyoshi, Tomonori Seo, Tadasu Matsuoka, Hiroko Itoh, Masayoshi Kurachi

**Affiliations:** ^1^Department of Neuropsychiatry, Graduate School of Medicine and Pharmaceutical Sciences, University of Toyama, 2630 Sugitani, Toyama 930-0194, Japan; ^2^Division of Molecular and Clinical Neurobiology, Department of Psychiatry, Ludwig-Maximilians University of Munich, Nußbaumstraße 7, 80336 Munich, Germany

## Abstract

The number of parvalbumin (PV)-positive **γ**-aminobutyric acid (GABA) neurons is decreased in the brain of rats transiently exposed to MK-801, an N-methyl-D-aspartate (NMDA) receptor blocker, in the neonatal stage (Uehara et al. (2012)). T-817MA [1-{3-[2-(1-benzothiophen-5-yl)ethoxy]propyl} azetidin-3-ol maleate] is a neuroprotective agent synthesized for the treatment of psychiatric disorders characterized by cognitive disturbances, such as dementia. We herein sought to determine whether T-817MA, haloperidol (HPD), or risperidone (RPD) would ameliorate the decrease in the number of PV-positive GABA neurons in the medial prefrontal cortex (mPFC) and hippocampus of the model animals. Rats were treated with MK-801 (0.2 mg/kg/day) or vehicle on postnatal days (PD) 7–10, and the number of PV-positive neurons in the mPFC and hippocampus were measured on PDs 63. T-817MA (20 mg/kg), HPD (1 mg/kg), or RPD (1 mg/kg) were administered during PDs 49–62. Fourteen-day administration of T-817MA reversed the decrease in the number of PV-positive neurons in the above brain regions of rats given MK-801, whereas HPD and RPD were ineffective. These results indicate that T-817MA provides a novel pharmacologic strategy to enhance cognitive function in patients with schizophrenia.

## 1. Introduction

Schizophrenia is a complex and severe disorder, which affects approximately 1% of general population [1, 2]. Patients with the illness manifest positive symptoms (e.g., delusions, hallucinations, thought disorder) and negative symptoms (e.g., anhedonia, blunted affect, social withdrawal), as well as disturbances in a range of cognitive domains, such as several types of memory, attention/information processing, executive functions, and verbal fluency [2]. The clinical course of schizophrenia is characterized by episodic positive symptoms and progressive negative and cognitive symptoms. The onset of illness is generally in the late adolescence or early adulthood, which is preceded by prodromal symptoms, including nonspecific mood symptoms, mild psychotic symptoms, cognitive impairment, and social withdrawal [3]. About one-third is resistant to treatment with existing antipsychotic drugs [4]. 

Schizophrenia is considered as a neurodevelopmental disorder [5, 6]. Progressive pathophysiological processes possibly begin in the prodromal stage and continue after the onset of the illness [3, 7, 8]. Apoptosis (programmed cell death) may play a role in this process that leads to neurodegeneration. The vulnerability of neurons to proapoptotic insults (proapoptotic triggers) could produce selective dendritic and synaptic losses [9]. The pro-apoptotic stimuli related to the pathophysiology of schizophrenia include (1) glutamatergic excitotoxicity, (2) excessive synaptic calcium flux, (3) oxidative stress, and (4) reduced neurotrophin levels (e.g., brain-derived neurotrophic factor BDNF, neurotrophin-3 NT-3) [9]. Thus, increased calcium levels and oxidative stress can lead to glutamate excitotoxicity and promote apoptotic activity [10]. Genetic and environmental factors, such as viral infection, may affect neural circuits during adolescence or young adulthood, leading to the emergence of positive and negative symptoms [11, 12]. Progressive volume reduction of whole brain, especially left and right prefrontal cortex, is found in individuals at high genetic risk of schizophrenia who later develop schizophrenia [13]. Therefore, there is increasing interest in the potential role for neuroprotection in the action of antipsychotic drugs [14]. Some second-generation antipsychotic drugs (SGAs) may have advantages in this respect, whereas the first-generation antipsychotic drugs, such as haloperidol particularly in high doses, may be neurodegenerative [14].

Dysfunction of *γ*-aminobutyric acid (GABA) interneurons, particularly those containing the calcium-binding protein parvalbumin (PV), has been suggested to be associated with the pathophysiology of schizophrenia, through the consequent imbalance between excitation and inhibition in the cerebral cortex [15, 16]. Abnormalities of GABA neurons are thought to be related to cognitive impairments of schizophrenia [15]. These considerations are consistent with the histological evidence for the reduction of PV-positive GABA interneuron density in the frontal cortex [15, 17, 18] and hippocampus [19, 20] in subjects with schizophrenia.

**Figure 1:**
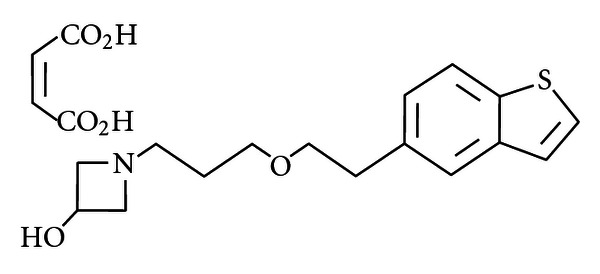
Chemical structure of T-817MA [1-{3-[2-(1-benzothiophen-5-yl) ethoxy]propyl} azetidin-3-ol maleate].

T-817MA [1-{3-[2-(1-benzothiophen-5-yl) ethoxy] propyl} azetidin-3-ol maleate] is a novel neuroprotective agent (Figure 1). It (1) exerts neuroprotective effects against neurotoxicity caused by intracerebroventricular infusion of amyloid-*β* (A*β*) [21, 22], (2) facilitates neurogenesis, such as neuron proliferation, neurite outgrowth, and synaptogenesis, through the increase of neurotrophic factors [23], and (3) improves cognitive impairment in rats receiving intracerebroventricular infusion of A*β* [21, 22] or expressing FTDP17 human P301L mutant tau [24]. T-817MA has been shown to also ameliorate behavioral and histological abnormalities in rodent models of schizophrenia [25, 26]. 

We previously reported that 14-day treatment with T-817MA ameliorated a decrease in the number of PV-positive GABA neurons in the medial prefrontal cortex (mPFC) and hippocampus of rats that received neonatal administration of MK-801, an N-methyl-D-aspartate (NMDA) receptor antagonist [25]. In this paper, we extend our studies by including haloperidol (HPD) and risperidone (RPD) as comparator compounds. 

## 2. Materials and Methods

### 2.1. Animals

Preparation of the animal model was based on previous reports [25, 27–29]. Female Wistar rats obtained at 14 days of pregnancy (Japan SLC, Hamamatsu, Japan) were housed individually at 24 ± 2°C under a light-dark cycle of 12:12 with lights onat 07:00 AM with free access to food and water. At the time of weaning (postnatal days; PD 21), the animals were grouped into four to six per treatment, in a cage with free access to food and water. The procedures complied with the National Institutes of Health Guide for the care and use of laboratory animals. All experiments were reviewed and approved by the Committee of Animal Research, University of Toyama. 

### 2.2. Neonatal MK-801 Treatment

On PD 7, male pups (7–15 g), born from 7 different female rats, were randomly divided into two groups; they received MK-801 (dizocilpine, 0.20 mg/kg, s.c., Sigma-Aldrich, St. Louis, MO; MK-801 neonatal treatment group) or an equal volume of saline (control; vehicle group) once daily for 4 days. Pups received injections between 8:00–10:00. 

### 2.3. Treatment with T-817MA, HPD, and RPD

The methods for administration of T-817MA were previously reported [25]. HPD and RPD were purchased from Dainippon Sumitomo Pharmaceuticals (Tokyo, Japan) and Janssen Pharmaceutical K. K. (Tokyo, Japan), respectively. On PD 49, animals were assigned to one of the following groups: saline-HPD group (*n* = 11), saline-RPD group (*n* = 10), MK801-HPD group (*n* = 10), and MK801-RPD group (*n* = 11). HPD (1.0 mg/kg) and RPD (1.0 mg/kg) were administered s.c. once daily (8:00–10:00) for 14 days 7 (PD 49–62). 

### 2.4. Immunohistochemical Study

#### 2.4.1. Fixation

On PD 63 (24 hours after the last treatment), rats were euthanized by deep anesthesia with sodium pentobarbital (Nembutal, Abbott Laboratories, USA) before transcardial perfusion fixation with saline followed by 4% paraformaldehyde in 0.1 M phosphate buffer. Brains were removed, postfixed in the same fixative at 4°C, and were stored in 30% sucrose solution at 4°C for cryoprotection. They were embedded in Tissue-Tek O.C.T. compound (Sakura Finetek USA, Torrance, CA, USA), rapidly frozen on dry ice, and were subsequently stored at −80°C. 

#### 2.4.2. Sectioning

Using a cryostat (Cryostats Leica CM 3050S, Leica Biosystems Nußloch GMBH, Nußloch, Germany), serial coronal sections of the brains were made with 30 *μ*m thickness through the mPFC and the hippocampus (AP from bregma 2.7 mm to −3.6 mm, resp.) [30]. The sections were mounted onto silage coated slides, dried for 4 hours at 38°C, and immersed in 0.1 M phosphate buffered saline containing 0.3% Triton X-100 (PBS-T) at 4°C.

#### 2.4.3. Immunostaining

Sections were incubated for 10 min in a solution of 0.6% hydrogen peroxide in 10% methanol to eliminate endogenous peroxidases. After three consecutive 5 min washes with PBS-T, they were blocked for 60 min in PBS-T containing 2% bovine serum albumin (Wako Pure Chemical Industries, Osaka, Japan). Then, the brain sections were incubated at 4°C overnight with a monoclonal anti-parvalbumin antibody 8 (Sigma-Aldrich, St. Louis) [1 : 3000 in antibody diluent (Dako, Glostrup, Denmark)]. After three consecutive 5 min washes with PBS-T, they were incubated for 60 min with horseradish peroxidase-labeled secondary antibody (Dako, Glostrup, Denmark). After washes with PBS-T, peroxidases were visualized using chromogen 3,3^'^-diaminobenzidine. 

#### 2.4.4. Quantification of Parvalbumin-Positive Cells

Quantification was performed in a blind fashion. All sections were observed using a light microscope (×10 objective, ECLIPSE E200, NIKON CORPORATION, Tokyo, Japan), and two sections which had intense signals of PV were analyzed for each brain area. PV-positive cell counts in the mPFC and hippocampus (whole part, as well as dentate gyrus, CA1, and CA2/CA3) were made bilaterally for two fields per section. The number of intensely stained neurons was counted within an 800 × 800 *μ*m^2^ area in the mPFC and granule cell layers in the hippocampal subfields. Pictures for the counting were acquired with a light microscope equipped with a digital camera (Microscope Digital System Moticam2000, SHIMADZU RIKA CORPORATION, Tokyo, Japan) and a software (Motic Image Plus 2.1S, SHIMADZU RIKA CORPORATION, Tokyo, Japan). Data was expressed as the number of intensely stained neurons per 1 mm^2^. 

### 2.5. Presentation of the Results and Statistics

Data were analyzed by analysis of variance (ANOVA) using SPSS software (version 19.0 J for Mac, IBM, Tokyo, Japan). Counts of PV-positive neurons were analyzed by one-way ANOVA followed by Bonferroni test.

## 3. Results

**Figure 2:**
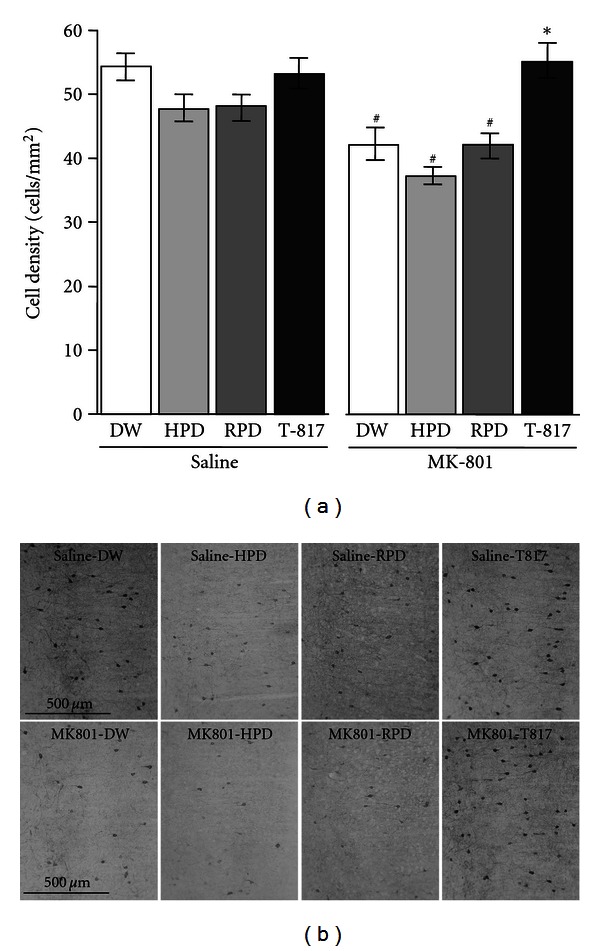
Effect of 14-day (PD 49–62) treatment with T-817MA, HPD or RPD on the density of PV-positive GABAergic interneurons (cells/mm^2^) in the mPFC. Data from DW group (*n* = 11), HPD group (*n* = 11), RPD group (*n* = 10), and T-817MA group (*n* = 11) treated neonatally with saline are shown in the left side. Data from DW group (*n* = 9), HPD group (*n* = 10), RPD group (*n* = 11), and T-817MA group (*n* = 9) treated neonatally with MK-801 are shown in the right side. Values are expressed as means ± SEM. ^#^; *P* < 0.05 as compared with saline-DW group. *; *P* < 0.05 as compared with MK-801-DW group. (b) Representative photomicrographs of PV-positive neurons in the mPFC from rats on PD 63.

The results of T-817MA have been already reported [25]. One-way ANOVA revealed significant differences in the count of PV-positive neurons among 8 groups (*F* = 8.74, *P* < 0.001). Post hoc Bonferroni test indicated that neonatal MK-801 treatment decreased PV-positive neurons (MK-801-DW group compared to saline-DW group, *P* = 0.007), which was ameliorated by T-817MA (MK-801-T817 group compared to MK-801-DW group, *P* = 0.006). HPD and RPD, on the other hand, did not influence the number of PV-positive neurons in MK-801-treated animals (MK-801-HPD group compared to MK-801-DW group, *P* = 1.00; MK-801-RPD group compared to MK-801-DW group, *P* = 1.00). Thus, HPD and RPD did not affect the number of PV-positive neurons in MK-801-treated animals in the mPFC, whereas T-817MA ameliorated its decrease (Figure 2(a)).

**Figure 3:**
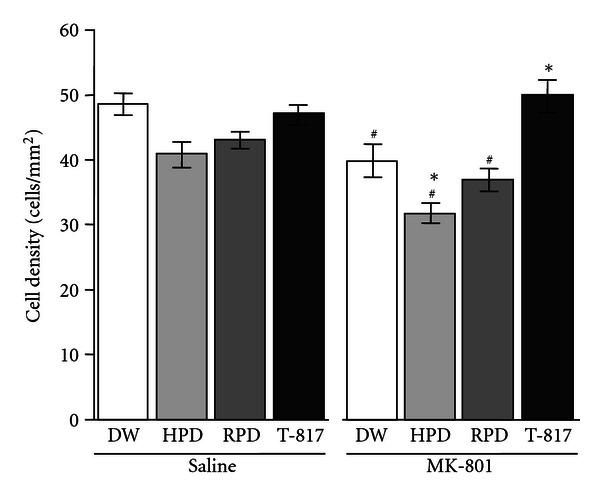
Effect of 14-day (PD 49–62) treatment with T-817MA, HPD, or RPD on the density of PV-positive GABAergic interneuron cells (cells/mm^2^) in the hippocampus in rats that did or did not receive neonatal treatment with MK-801. DW group (*n* = 12), HPD group (*n* = 10), RPD group (*n* = 10), and T-817MA group (*n* = 11) treated neonatally with saline are shown in the left side. DW group (*n* = 9), HPD group (*n* = 11), RPD group (*n* = 11), and T-817MA group (*n* = 9) treated neonatally with MK-801 are shown in the right side. Data of saline group (open bars), HPD group (dark bars), RPD group (shaded bars), and T-817MA group (closed bars) are shown. Values are expressed as means ± SEM. ^#^; *P* < 0.05 as compared with saline-DW group. *; *P* < 0.05 as compared with MK801-DW group.

In the hippocampus, one-way ANOVA demonstrated significant differences in the count of PV-positive neurons among 8 groups (*F* = 13.54, *P* < 0.001). Post hoc Bonferroni test showed that neonatal MK-801 treatment also decreased PV-positive neurons (MK801-DW group compared to saline-DW group, *P* = 0.016), which was reversed by T-817MA (MK801-T817 group compared to MK801-DW group, *P* = 0.006). By contrast, HPD enhanced the MK-801-induced decrease in the number of PV-positive neurons (MK801-HPD group compared to MK801-DW group, *P* = 0.042) (Figure 3(a)). 

**Figure 4:**
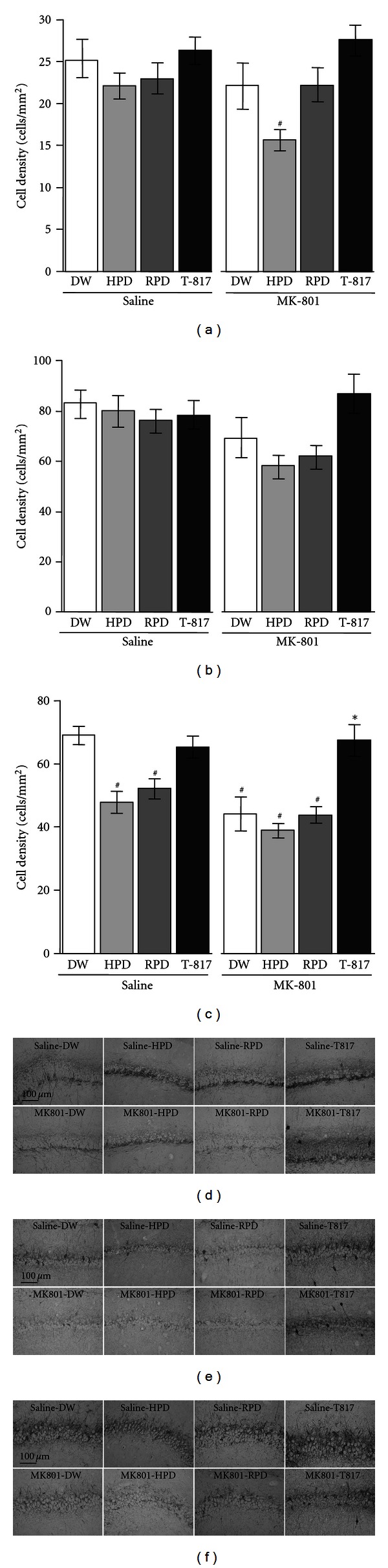
(a–c) Effect of 14-day (PD 49–62) treatment with T-817MA, HPD, or RPD on the density of PV-positive GABAergic interneuron cells (cells/mm^2^) in the hippocampal subfields, that is, DG (a), CA1 (b), and CA2/3 (c), in rats that did or did not receive neonatal treatment with MK-801. DW group (*n* = 12), HPD group (*n* = 10), RPD group (*n* = 10), and T-817MA group (*n* = 11) treated neonatally with saline are shown in the left side. DW group (*n* = 9), HPD group (*n* = 11), RPD group (*n* = 11), and T-817MA group (*n* = 9) treated neonatally with MK-801 are shown in the right side. Data of saline group (open bars), HPD group (dark bars), RPD group (shaded bars) and T-817MA group (closed bars) are shown. Values are expressed as means ± SEM. ^#^; *P* < 0.05 as compared with saline-DW group. *; *P* < 0.05 as compared with MK801-DW group. (d)–(f) Photomicrographs of PV-positive neurons in subregions of hippocampus from rats on PD 63; DG (d), CA1 (e), and CA2/3 (f).

We subsequently analyzed the number of PV-positive neurons in the subregions of the hippocampus (DG, CA1, CA2/3 areas). In DG, one-way ANOVA revealed significant differences among 8 groups (*F* = 3.15, *P* = 0.003) (Figures 4(a) and 4(d)). Post hoc test showed that PV-positive neurons were decreased in MK801-HPD group compared with saline-DW group (*P* = 0.005). In CA1, the number of PV-positive neurons was significantly different among the 8 groups (*F* = 3.11, *P* = 0.006), due to a larger number of cells in MK801-T817 group compared to MK801-HPD group (*P* = 0.031). On the other hand, post-hoc analysis revealed none of the MK-801 or antipsychotic-treated groups was significantly different from saline-DW group or MK801-DW group (Figures 4(b) and 4(e)). There was a significant treatment effect in the CA2/3 area (one-way ANOVA; *F* = 12.05, *P* < 0.001). Post hoc test demonstrated that neonatal MK-801 treatment decreased PV-positive neurons (MK801-DW group compared with saline-DW group, *P* < 0.001). T-817MA reversed the MK-801-induced decline in the number of PV-positive neurons (MK801-T817 group compared with MK801-DW group, *P* = 0.001). HPD and RPD, with or without combination with neonatal MK-801 treatment, decreased PV-positive neurons (in comparison with saline-DW group; *P* < 0.02) (Figures 4(c) and 4(f)).

## 4. Discussion

The results of this study demonstrate the ability of T-817MA to ameliorate the reduction of PV-positive GABA neurons in the brain of rats transiently exposed to NMDA receptor blockade at the neonatal period. On the other hand, both HPD and RPD did not show such effect in the mPFC of the neurodevelopmental model animals. In DG, PV-positive neurons were decreased by the combination of neonatal MK-801 administration and HPD treatment, compared with other treatment regimens. These model rats showed a decrease in the number of PV-positive neurons in the CA2/3, but not CA1 subfields, which was reversed by T-817MA. HPD and RPD by themselves decreased PV-positive neurons in rats with or without neonatal exposure to MK-801. 

Treatment with HPD, RPD, or T-817MA was started around the period of puberty (PD 49 to 62) in our model animals. This timing was chosen based on the observations that (1) patients with schizophrenia manifest psychosis around puberty [1, 2], and (2) our model rats elicit disruption of sensorimotor gating and increased methamphetamine-induced locomotor activity at postpuberty (PD 63), but not prepuberty (PD 35) [28, 29].

### 4.1. Effect of T-817MA

T-817MA elicits neuroprotective effects against amyloid-*β* or H_2_O_2_-induced neurotoxicity, while decrease of glutathione (GSH) levels induced by H_2_O_2_ exposure was suppressed by pretreatment with T-817MA [23]. GSH is an important intracellular antioxidant that protects the neurons against a variety of reactive oxygen species (ROS) [31]. In addition, this agent reduced attenuation of ROS production in mitochondria [32]. T-817MA has been also shown to facilitate neurogenesis in vitro. Thus, it promotes neurite outgrowth and increases the amount of growth-associated protein 43 in hippocampal slice cultures and neuronal reaggregation culture [23]. T-817MA has been also demonstrated to increase PSA-NCAM, a marker of cell proliferation, and bromodeoxyuridine (BrdU)-positive cells in the dentate gyrus (DG) of rats that received continuous A*β* infusion into the cerebral ventricles [22]. These findings indicate that T-817MA stimulates proliferation of neural progenitor cells and enhances survival of the newly generated cells in the DG against neurotoxicity [22].

Behaviorally, T-817MA has been shown to ameliorate memory impairment in rats receiving intracerebroventricular infusion of A*β* [21, 22]. Thus, T-817MA has been demonstrated to improve cognitive impairment, as measured by the Y-maze task, in mice expressing FTDP17 human P301L mutant tau [24]. This agent also corrects tau-induced synaptic abnormalities and enhances synaptic terminal density in the hippocampus [24]. 

The protective effect of T-817MA against human tau (h-tau42)-mediated axonal/synaptic dysfunction has been demonstrated by means of tau-mediated synaptic block, synaptic vesicle aggregation, and decreased h-tau42 phosphorylation [33]. Moreover, T-817MA has been suggested to protect against Parkinson's disease. For example, it can prevent 1-methyl-4-phenyl-1,2,3,6-tetrahydropyridine (MPTP)-induced dopaminergic neurotoxicity in C57BL/6J mice [34]. MPTP impairs mitochondrial respiration by inhibiting complex 1 and causes dopaminergic neurotoxicity leading to behavioral impairment similar to the features of Parkinson's disease [35, 36]. Pretreatment with T-817MA attenuates MPTP-induced decrease in dopamine levels and tyrosine hydroxylase immunostaining in the substantia nigra (SNc) and striatum [34]. It also ameliorates impairment of rotarod performance, a measure of coordinated motor skills [34]. Taken together, T-817MA is thought to exert neuroprotective effects against neurotoxicity caused by antioxidative insults. 

### 4.2. Effects of HPD and RPD

Some researchers report the effects of HPD or RPD on decreased PV-positive neurons induced by NMDA antagonists [37, 38]. Thus, coadministration of HPD attenuated the detrimental effect of MK-801 on PV-positive neurons in the hippocampus of juvenile rats, but markedly reduced immunoreactivity to PV in the prefrontal cortex [37]. Concurrent administration of RPD with PCP did not protect against reduction in the expression of PV-positive neurons in the prefrontal cortex [38]. These previous findings are partly consistent with the results of our data (Figure 2(a)). Moreover, it is reported that pretreatment with olanzapine prevented the apoptosis in the frontal cortex of rat induced by 10 mg/kg PCP administration on PD 7, 9, and 11 [39]. This finding suggests that perinatal administration of some SGAs may block apoptosis. 

In sum, some of the existing antipsychotic drugs, such as HPD and RPD, show the limited ability to protect PV-positive interneurons in the mPFC and hippocampus in animals that received transient excitotoxic insults in the neonatal period.

## 5. Conclusion 

Using a neurodevelopmental animal model, we have demonstrated the ability of T-817MA to ameliorate histological abnormalities in brain areas responsible for cognitive disturbances of schizophrenia, while some widely used antipsychotics did not show such effect. To our knowledge, T-817MA is the first agent to reverse the reduction in the number of PV-positive GABA interneurons induced by blockade of NMDA receptors. Our findings may provide a novel approach for the treatment of the core features of schizophrenia, such as cognitive deficits.
